# The Kilovoltage Intrafraction Monitoring Real-Time Prostate Cancer Image Guided Radiation Therapy Technology Journey: From Clinical Trials to Your Clinic

**DOI:** 10.1016/j.ijrobp.2026.01.010

**Published:** 2026-01-18

**Authors:** Paul J. Keall, Jeremy Booth, Thomas Eade, Emily A. Hewson, Amar U. Kishan, Michael Jameson, Kankean Kandasamy, Andrew Kneebone, Doan Trang Nguyen, Per Rugaard Poulsen, Chandrima Sengupta, Daniel E. Spratt, Alison Tree, Pengpeng Zhang, Jarad Martin

**Affiliations:** a Image X Institute, University of Sydney, Sydney, New South Wales, Australia;; b Department of Radiation Oncology, Northern Sydney Cancer Centre, Royal North Shore Hospital, Sydney, New South Wales, Australia;; c Department of Radiation Oncology, University of California Los Angeles, Los Angeles, California;; d University of California Jonsson Comprehensive Cancer Center, Los Angeles, California;; e GenesisCare, Alexandria, New South Wales, Australia;; f School of Clinical Medicine, University of New South Wales, Sydney, New South Wales, Australia;; g Centre for Medical Radiation Physics, University of Wollongong, Wollongong, New South Wales, Australia;; h SeeTreat, Sydney, New South Wales, Australia;; i Danish Centre for Particle Therapy, Aarhus University Hospital, Aarhus, Denmark;; j Department of Clinical Medicine, Aarhus University, Aarhus, Denmark;; k Department of Radiation Oncology, UH Seidman Cancer Center, Cleveland, Ohio;; l University Hospitals at Case Western Reserve University, Cleveland, Ohio;; m Department of Urology, The Royal Marsden NHS Foundation Trust and The Institute of Cancer Research, Sutton, United Kingdom;; n Department of Medical Physics, Memorial Sloan Kettering Cancer Center, New York, New York;; o Department of Radiation Oncology, Calvary Mater, Newcastle, New South Wales, Australia;; p Institute of Medical Physics, School of Physics, University of Sydney, Sydney, Australia

## Abstract

**Purpose:**

Stereotactic Body Radiation Therapy (SBRT) schedules for prostate cancer are emerging as a standard treatment approach. However, although high efficacy is achieved in clinical trials, additional efforts at accurate treatment delivery are necessary to maximize the therapeutic ratio in the community setting. Kilovoltage Intrafraction Monitoring (KIM) is a technology developed to address the unmet clinical need of accurate intrafraction motion management without a requirement for additional hardware on standard linear accelerators. This paper describes the journey from bench to bedside of KIM.

**Methods and Materials:**

The concept of KIM emerged in 2008 in a series of simulations, progressing to phantom experiments commencing in 2010, culminating in the first clinical trial starting in 2014. A series of technical innovations integrating rotational prostate movements, quality assurance, and dynamic multileaf collimator tracking were included within the multi-institutional Trans-Tasman Radiation Oncology Group 15.01 Stereotactic Prostate Adaptive Radiation therapy using KIM clinical trial for prostate SBRT.

**Results:**

Submillimeter accuracy of KIM was observed in simulations, phantom experiments, and clinical trials. The Stereotactic Prostate Adaptive Radiation therapy using KIM trial demonstrated multicenter feasibility of use, a high rate of intrafraction motion triggering a gating event, and a significant dosimetric impact of KIM deployment for target volume coverage. KIM is being made available through commercialization with an industry partner, and through a 300-patient, 10-center clinical trial.

**Conclusions:**

The collaboration between researchers, clinicians, and industry has led to KIM being developed from a concept to clinical trials, enabling the safe delivery of prostate cancer SBRT in the presence of intrafraction motion. This journey provides a template for similar bench-to-bedside translational research.

## Introduction

Stereotactic body radiation therapy (SBRT) offers therapeutic benefit but also risks. This risk is high in dynamic sites such as the prostate, where changes in rectal and bladder volumes in real time can affect target positioning. The longer treatment times associated with SBRT regimens increase the potential for intrafraction movements and deformations, which can inflict an adverse dosimetric impact. Although multiple solutions exist to address intrafraction motion, most require expensive additional hardware, which can be challenging to integrate into standard treatment in the community. This paper describes the evolution of kilovoltage intrafraction monitoring (KIM) as a real-time image guided radiation therapy (IGRT) technology.

KIM is a medical device that tracks the internal target motion of implanted fiducial markers in 3 dimensions from x-ray images in cancer radiation therapy (RT). This target motion can be used to facilitate gating events, inform couch shifts, adjust the treatment beam, and provide continuous target positional information to its operators.

The context of the real-time IGRT technology development was an increasing awareness of, and emerging tools to manage, motion.^1^ As early as 2008 American Society for Radiation Oncology guidelines stated *A precise ability to localize the target tumor is essential to fully benefit from SBRT techniques*.^2^ Real-time RT,^3^ ultrasound,^4,5^ robotic x-ray,^6^ and electromagnetic^5–8^ solutions were being introduced. The KIM technology concept differed from these other solutions as KIM used existing linear accelerator equipment, ie, a single gantry-mounted x-ray imager, to determine the 3-dimensional (3D) target position, overcoming the need for additional hardware. The demand for cost-effective and integrated real-time IGRT technology has been quantified—an European Society for Radiotherapy and Oncology survey of 200 respondents in 40 countries found that 71% wish to implement real-time IGRT technology for additional cancer sites, but were limited by resources and availability.^9^

**Fig. 1.**
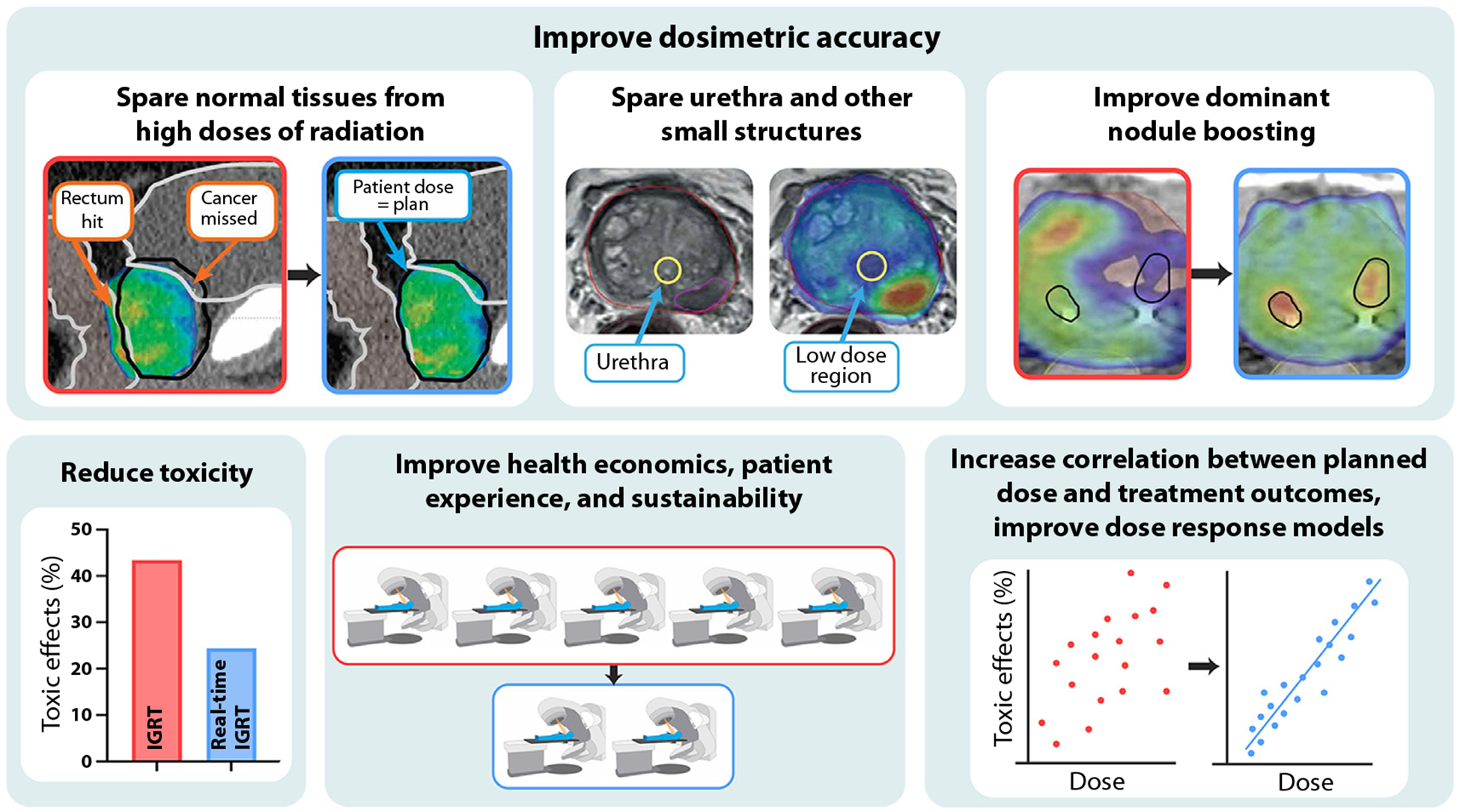
Clinical drivers for real-time IGRT technology. *Abbreviation*: IGRT: image guided radiation therapy.

Clinical drivers for real-time IGRT, expanded on in the discussion and shown in Figure 1, include the ability to:

- reduce toxicity,^10–12^
- reduce dose to rectum, bladder, urethra, neurovascular bundles, and other small structures,^13–16^
- improve dominant nodule boosting,^17^
- enable fewer fraction treatments to improve health economics, the patient experience, and sustainability,^18,19^
- increase the correlation between dose delivered and treatment outcomes, and improve data for dose-response modeling.^20–24^

As a medical device, KIM has the following value propositions for radiation oncology:

- Reduce uncertainty in patient treatments: directly linked to the aforementioned clinical drivers. KIM is directly monitoring internal target motion which is a more direct surrogate for tumor motion compared with external (or indirect) surrogates.
- Efficient treatment: KIM can confirm patient alignment during treatment without the need for multiple cone beam computed tomography scans, reducing time and radiation dose. A planned clinical version of KIM with linear accelerator integration, allowing automatic gating and integrated couch shift, makes the motion adaptation process efficient.
- Minimal overheads: No additional equipment or alterations to clinical setup. KIM enables enhancement to a site’s existing clinical setup for standard linear accelerators. KIM requires no additional disposables or equipment.
- Minimizing unnecessary imaging dose: KIM’s couch shift information negates the need for additional positioning cone beam computed tomography images.

This paper describes the simulation, experimental, clinical trials, and commercial journey of taking a technology from a concept to a product. The near 20-year timescale of scientific discovery, academic development, intensive clinical trial completion, regulatory compliance, and industry engagement is described. This experience of 1 device aims to assist other clinicians and scientists in understanding and navigating the challenges of taking a technical innovation from conception through the full translational pathway.

## Methods and Materials

**Fig. 2.**
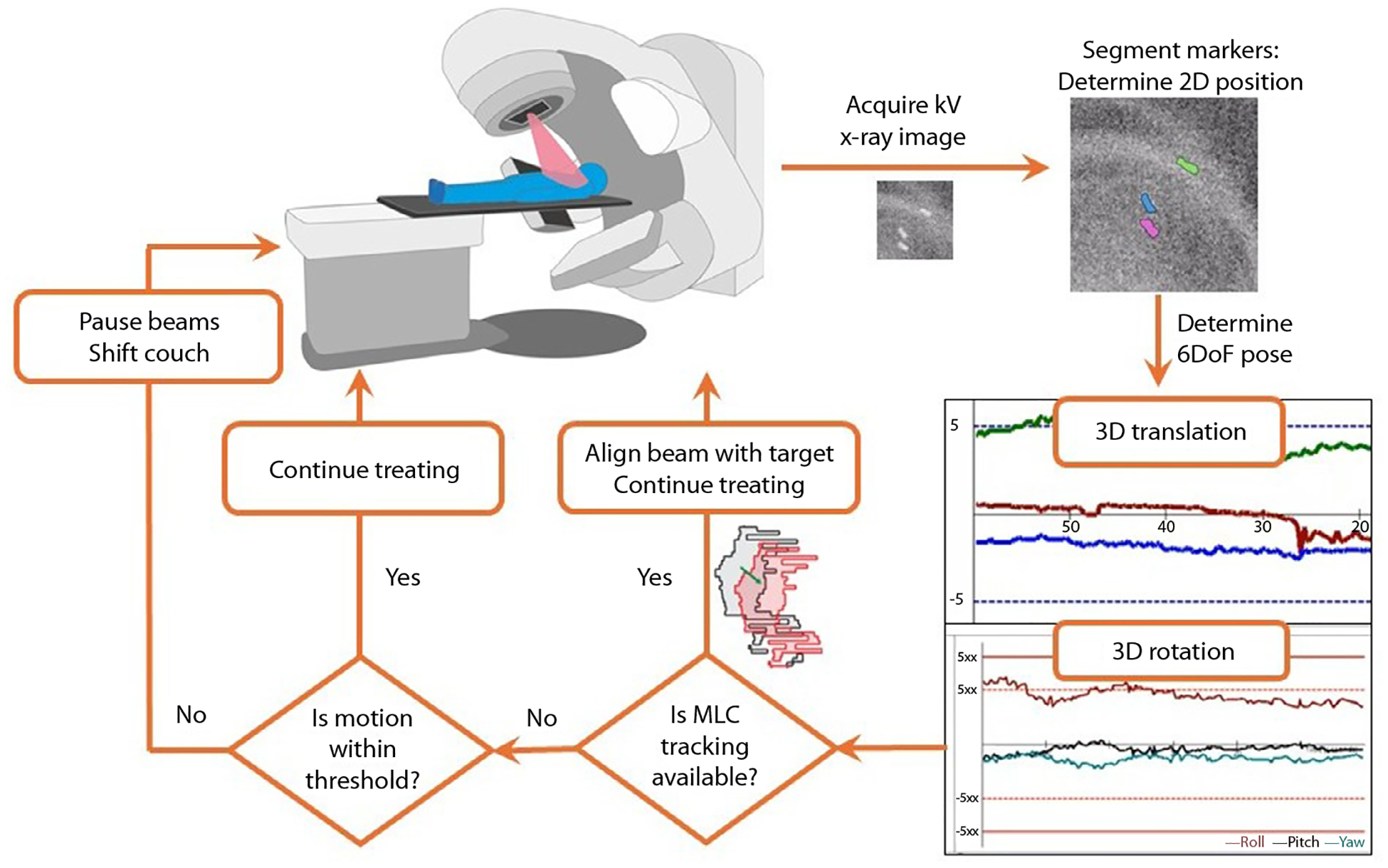
The KIM real-time IGRT technology workflow. Where multileaf collimator (MLC) tracking is not available, gating is used with a predefined threshold, typically ±2 mm for SBRT. *Abbreviations:* DoF: degrees of freedom; IGRT: image guided radiation therapy; KIM: Kilovoltage Intrafraction Monitoring; SBRT: stereotactic body radiotherapy.

KIM is an IGRT method that estimates 3D translational (and more recently rotational) target motion in real time from a sequence of x-ray images acquired at different angles by a single imager. The concept of KIM started in 2007 with the question, “How can you obtain real-time 3D information from sequential 2D images?” A mathematical solution, to combine prior and real-time images via a maximum likelihood framework, was published in 2008^25^ and sparked a large research endeavor resulting in the first patient treatment in 2014.^26^ This clinical milestone was followed by further trials, including the Trans-Tasman Radiation Oncology Group (TROG) 15.01 Stereotactic Prostate Adaptive Radiation therapy using Kilovoltage Intrafraction Monitoring (SPARK) prostate cancer SBRT trial.^13^ A functional diagram of KIM is shown in Figure 2.

**Fig. 3.**
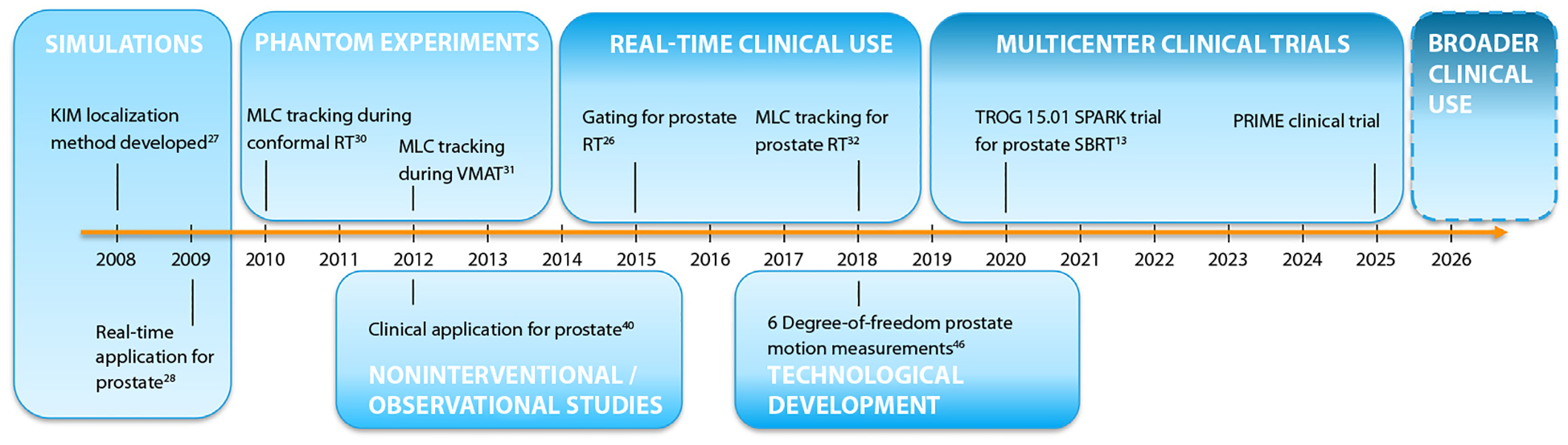
Timeline of the KIM real-time IGRT technology journey. *Abbreviations:* IGRT: image guided radiation therapy; KIM: Kilovoltage Intrafraction Monitoring; MLC: multileaf collimator; PRIME: Prostate Radiotherapy Therapy kilovoltage intrafraction monitoring Real-World Expansion; RT: radiation therapy; SBRT: stereotactic body radiation therapy; SPARK: Stereotactic Prostate Adaptive Radiation therapy using kilovoltage intrafraction monitoring; TROG: Trans-Tasman Radiation Oncology Group; VMAT: volumetric modulated arc therapy.

Figure 3 shows the timeline of the KIM real-time IGRT technology journey with the dates of key publications. So far, nearly all studies used a 2-step 3D localization method that first fits a 3D normally distributed probability density function (PDF) to the 2D target positions in the image sequence by maximum likelihood estimation and then estimates the 3D position for individual images as the most probable position along the line-of-sight according to the PDF.^27,28^ A Kalman filter method provides an optimal method to estimate the 3D position and update the PDF at the same time.^29^ This approach yields submillimeter accuracy by implicitly detecting and exploiting motion correlation along different axes and motion confinement along a line or in a plane. KIM can be performed in real time using intratreatment images by continuously updating the PDF each time a new image is acquired and then performing the 3D localization from the newest image.^28^ This approach allowed the first image guided multileaf collimator (MLC) tracking during conformal^30^ and volumetric modulated arc therapy in phantom experiments^31^ and was later translated to the clinic for gating^26^ and MLC tracking^32^ during prostate SBRT. There is a major step from phantom experiments to real-time clinical interventional application, where imaging dose must be considered^33,34^ and extensive quality assurance^35^ is needed to mitigate the risk of treatment errors caused by erroneous real-time localization. Alternative methods for 3D localization include the use of motion correlation along different axes for respiratory motion,^36^ and triangulation with previous images from different angles for stable or recurrent motion.^37^

### The first KIM trial

In 2014, after 7 years of preclinical research, the first patient was treated with KIM for prostate cancer RT. The trial (NCT01742403) accrued 30 patients with biopsy-confirmed prostate cancer from stage T1c to T2 to 80 Gy in 2 Gy fractions between 2014 and 2016.

### The multi-institutional SPARK trial

The first trial was followed by the prospective, multicenter, phase 2 TROG 15.01 SPARK clinical trial.^13^ This trial recruited patients with low to intermediate risk prostate cancer to be managed with SBRT, with the primary outcome being whether intrafraction repositioning using KIM would meaningfully impact delivered dose distributions compared with a nonadaptive approach.

### The clinical integration of KIM-guided MLC tracking

Building on the integration of Calypso-guidance with MLC tracking,^17,38^ KIM-guided MLC tracking was evaluated and clinically implemented in 10 patients treated as part of the TROG 15.01 SPARK trial. A differentiator from the Calypso-guided MLC tracking studies is that KIM-guided MLC tracking requires only on-board kilovoltage (kV) imaging and software to deliver real-time adaptive RT tracking of implanted fiducials in the prostate.

### KIM geometric accuracy

To evaluate the geometric accuracy of KIM during patient treatments, the marker positions determined by KIM were retrospectively analyzed using kV-megavoltage (MV) triangulation. During each patient treatment, MV images were obtained using the electronic portal imaging device with acquisition rates ranging from 7.5 to 10 Hz.^39,40^ The groundtruth 3D position was determined by triangulating the marker positions in the orthogonal kV and MV images and was compared with the 3D position determined by KIM. The geometric accuracy (mean error) and precision (SD of errors) were calculated by taking a vector difference between the 2 positions. For MV images where all 3 markers could be segmented, the rotation of the prostate was also calculated and compared with the rotation estimated by KIM during treatment.

### Estimation of the delivered dose via dose accumulation

The measurement of real-time target motion enables an estimate of the dose delivered to the dynamic anatomy during treatment. One method of dose accumulation to obtain dose to the target and organs at risk (OARs) in the presence of motion is to use a time-resolved isocenter shift method.^41^ This method has been widely adopted for KIM prostate cancer treatments to date. To compute the dose to the patients in simulated treatments without KIM, the KIM-measured prostate motion without couch corrections was used as the input to the dose accumulation method. To quantify the dosimetric impact of observed intrafraction rotation of the prostate measured using KIM, a dose grid resampling algorithm was used,^42^ and also software that shifted each individual calculation point between consecutive dose increment calculations according to KIM-measured motion and calculated dose in each point.^43^

One main assumption in dose accumulation used in these studies^41^ is that the high-dose region in the bladder and rectum moves rigidly with clinical target volume (CTV) around the rotation centroid. This assumption is justified to first order as the dose gradient is steep, so that the high-dose regions rotate together around the same centroid. A second limitation of these studies is that the uncertainties related to target definition and marker localization were not considered. The dose metrics analyzed were the dose to 98% of the CTV (CTV D98%) and the bladder and rectum volume receiving >30 Gy (V30Gy).

## Results

### The first KIM trial

The KIM Gating trial (NCT01742403) included the first-in-human use of KIM.^26^ The imaging and motion information from the first 200 fractions from 6 patients with prostate cancer were analyzed.^44^ The clinical implementation of KIM-guided gating for prostate cancer eliminated large prostate displacements during treatment delivery. Three millimeter per 5 second gating events occurred in 29 of the first 200 fractions (14.5%). In these 29 fractions, prostate displacement from the isocenter position >3 mm was reduced from 73% to 24% by KIM Gating. Displacement >5 mm was reduced from 16% without KIM to 0% with KIM. The KIM accuracy was measured to be <0.3 mm in all 3 dimensions. The KIM precision was <0.6 mm in all 3 dimensions.^44^

### The multi-institutional SPARK trial

The TROG 15.01 SPARK trial accrued 48 patients from 5 centers between 2016 and 2018.^13^ First, the deployment of KIM was technically feasible, with all 3 translational and rotational axes trackable in real time on both Elekta and Varian linacs. Next, 88% (42/48) patients had motion correction for at least 1 treatment, with 51% of fractions requiring a treatment interruption and repositioning. The typical tolerance for motion correction exceeded 2 mm of motion in any direction for 5 seconds. Regarding the primary endpoint, with KIM, the prostate CTV D98% was within 5% of the original plan for all 240 fractions. Conversely, 13 of the 240 fractions were estimated to have reduced target volume coverage in the absence of KIM. Clinically, no changes were observed in patient-reported quality of life at 12 months. The physician graded toxicity was low, with no grade ≥3 genitourinary or gastrointestinal toxicities reported.

### The clinical integration of KIM-guided MLC tracking

The integration of KIM-guided MLC tracking in the TROG 15.01 SPARK trial was successful.^32^ KIM-guided MLC tracking demonstrated comparable delivered dose with KIM-guided gating^45^ with increased treatment efficiency.

### KIM geometric accuracy

KIM quality assurance processes using programmable motion platforms were performed across all institutions and linear accelerator platforms for static localization, dynamic localization, and treatment interruption accuracy. Across these tests in all institutions, the mean and SD of the KIM prediction and ground truth were <1.0 mm, and the measured latency was 350 ms.^35^

On the patient data, the geometric accuracy and precision of KIM was within 1 mm for all clinical implementations of KIM for prostate treatments.^39,40,44,46^ Initial clinical implementation of KIM found a 3D error of 0.4 ± 0.5 mm^40^ and errors in the left-right (LR), superior-inferior (SI), and anterior-posterior (AP) directions of −0.3 mm ± 0.5 mm, 0.1 mm ± 0.4 mm, and 0.1 ± 0.5 mm, respectively.^44^ Later clinical implementations of KIM for prostate also estimated rotation in addition to translation and measured rotational errors of −0.2° ± 1.0°, 0.0° ± 1.3°, −0.1 ± 0.5° around the LR, SI, and AP axes, respectively,^46^ A similarly high geometric accuracy and precision was maintained when KIM was used for SBRT in the TROG 15.01 SPARK trial.^39^ No correlation between geometric error and the magnitude of motion has been observed.^39,44,46^ Geometric accuracy was seen to be highest in the directions that are resolved in the imaging plane, so a small imaging angle dependency has been observed for directions that are not resolved at all imaging angles.^39,46^

These results were analyzed for 5 institutions using 3 linear accelerator models (Varian TrueBeam, Varian Trilogy, and Elekta) and 2 beam models (6 MV, 10 MV flattening filter free). Correlations between geometric error and motion magnitude and marker correlation all remained <0.44 across institutions, with no substantial variability in accuracy or precision between institutions.^39^ These findings suggest that KIM can be implemented reliably across different linear accelerator platforms and institutions.

### Estimation of the delivered dose via dose accumulation

To put the dosimetric results into context, a 5% dose difference between the planned dose and that delivered to the patient has been considered clinically meaningful.^47^ Considering translation-only motion in the dose accumulation in the SPARK study, the number of fractions with the prostate CTV dose 5% less, or the rectal or bladder dose 5% more than the planned dose was 0, 0 and 0 (out of 240 total fractions), respectively. In simulated treatments without KIM, the number of treatments with the prostate CTV dose 5% less, or the rectal and bladder dose 5% more than the planned dose was 13, 4 and 14 (out of 240), respectively. The prostate CTV D98% dose with KIM was closer to the plan than without KIM by an average of 1.0% (range, −2.8% to 20.3%). The rectum V30Gy dose with KIM was closer to the plan than without KIM in 86% of the treatments by an average of 1.5% (range, −1.2% to 9.7%). The bladder V30Gy dose with KIM was closer to the plan than without KIM in 90% of the treatments by an average of 1.8%, with the range from −2.3% to 14%.^13^

Including both intrafraction translation and rotation in the dose accumulation, the dose to CTVD98% was always within 5% of the plan delivered with KIM; however, it was greater than 5% in 1 fraction for bladder V30Gy and in 5 fractions for rectum V30Gy.^42,43^ These studies indicate that residual errors due to uncorrected rotation exist, which may be corrected with different treatment adaptation techniques to further improve dosimetric accuracy.

### Kilovoltage x-ray imaging dose

Relevant to KIM and other x-ray–based guidance technologies, recommendations for managing and reducing imaging dose are given by American Association of Physicists in Medicine (AAPM) Task Group report 180.^48^ The SPARK clinical trial found the additional dose to the prostate was at most 1.3% of the total dose.^49^ This value is below the 5% recommended threshold beyond which imaging dose should be considered in the treatment planning process.^48^ Several methods to reduce the imaging dose from KIM have been identified, including reducing the imaging frequency, using patient and gantry angle-specific kV field sizes, improving the marker detection software to allow lower dose per image, and implementing automatic exposure control.^40^

## Discussion

One of the central tenets for safe and effective SBRT is accurate treatment delivery. A major paradigm shift that made this possible was the introduction of daily pretreatment IGRT. However, significant residual position errors can still occur due to intrafraction motion. Although bespoke platforms exist to manage intrafraction motion, most require dedicated hardware and significant financial commitments. This paper describes the KIM development pathway where a software solution to enable real-time 3D IGRT has proven to be technically deliverable, results in more accurate position and delivered dosimetry, and can be integrated with gating and MLC tracking.

The following 6 subsections expand on the clinical benefits of real-time 3D IGRT. As KIM is a potentially widely available real-time 3D IGRT technology, these benefits can be realized with KIM, although the benefits pertain to other accurate real-time 3D IGRT technologies. Each real-time IGRT technology will have different advantages and disadvantages. Two notable disadvantages of KIM compared with both magnetic resonance imaging^10,11,50^ (MRI)and ultrasound-guided approaches^4,5,50^ are the addition of x-ray imaging dose, described in the final section of the Results above, and the inability to directly image the urethra and other structures. Instead, their locations are inferred based on their relationship to implanted markers from the treatment plan. Additional limitations include the frame rate of the x-ray imaging, which depends on a tradeoff between increased temporal resolution and reduced imaging dose, potential marker migration, and the inability to directly measure anatomical deformation.

### Reduced toxicity with real-time 3D IGRT technology

The phase III CT-guided Stereotactic Body Radiation Therapy and MRI-guided Stereotactic Body Radiation Therapy for Prostate Cancer MIRAGE trial (NCT04384770) provided randomized trial evidence of reduced toxicity with real-time IGRT technology.^11^ Major contributors to the uncertainty underlying the planning target volume (PTV) margin in prostate cancer RT are intrafraction motion,^51,52^ residual error from MRI-CT fusions in the contouring process, and setup uncertainties during pretreatment rigid registration.^53^ MRI-guided linear accelerators can mitigate these uncertainties with real-time motion tracking of the prostate directly with cine MRI imaging, reliance on MRI for contouring, and enhanced soft-tissue contrast during rigid registration, respectively. In the MIRAGE trial, patients were randomized to receive either CT-guided prostate SBRT with standard-of-care 4-mm PTV margins or MRI-guided gated prostate SBRT with 2-mm PTV margins.^11^ The trial demonstrated a significant reduction in acute grade ≥2 genitourinary toxicity (43.4% vs 24.4%; primary endpoint) and acute grade ≥2 gastrointestinal toxicity (10.5% vs 0%).^11^ A 2-year update confirmed reduction in late grade ≥2 genitourinary toxicity (51% vs 27%) and late grade ≥2 gastrointestinal toxicity (9.5% vs 1.4%).^10^ Patient-reported quality-of-life metrics were improved as well. A post-hoc analysis of the trial confirmed the dosimetric improvement of gating in mitigating intrafraction motion.^54^ A successor trial is evaluating toxicity following CT-guided SBRT with 2-mm margins, with margin reduction achieved with triggered imaging and automatic beam hold or adaptive therapy (NCT06995053). Although there are differences between real-time MRI-guided and x-ray marker-based IGRT, the toxicity reductions observed with margin reduction in the MIRAGE trial are hypothesized to also be achievable by KIM and related technologies. A key additional consideration then becomes cost effectiveness, particularly given the extra hardware required for an MRI-Linac or even intraprostatic transponders, versus a software-based solution such as KIM. Any hypothesis should be rigorously tested in prospective clinical trials.

In parallel to the development and clinical implementation of KIM, a unique MV-kV image guidance system that uses both MV and kV imagers mounted on a modern linear accelerator has been developed. The MV-kV system periodically acquires orthogonal MV and kV image pairs during volumetric modulated arc therapy delivery and localizes 3 prostate-implanted fiducials via template matching with the planning CT and 3D triangulation. The real-time 3D prostate motion is used to gate the beam delivery to ensure proper dose coverage. This automated and quantitative MV-kV system has been demonstrated to introduce minimal workflow impact and correlate with a clinically significant reduction in late urinary toxicity in a retrospective study of 271 prostate SBRT patients.^12^ Both KIM and MV-kV systems provide clinically proven and robust image guidance for prostate SBRT. Further investigations, such as reducing clinical margins, are warranted to maximize the clinical benefit of intrafraction motion management with these systems and provide evidence for future randomized clinical trials.

Other real-time IGRT approaches that have been developed and clinically implemented using existing linear accelerator equipment include SeedTracker^55,56^ and Triggered Imaging.^57^ Both SeedTracker and Triggered Imaging give 2D positional information in the imager frame of reference rather than 3D. However, there is no limitation to extending these approaches to enable real-time 3D IGRT, as several 2D to 3D methods have been published.^28,29,36,37^

### Reduced dose to rectum and bladder

The MIRAGE trial^11^ demonstrated that the implementation of real-time IGRT enables reduced CTV to PTV margins, in that trial from 4 to 2 mm, thereby reducing the dose to the rectum and bladder. An analysis of the SPARK trial results with KIM found that for the same margin, with real-time IGRT compared with no real-time IGRT, the rectum and bladder doses were more consistent from fraction to fraction, with fewer instances of doses significantly exceeding the planned value.^13^

### Reduced dose to urethra, neurovascular bundles, and other small structures

Ultraprecise dose delivery is critical in the presence of small OAR substructures whose function can be impacted with changes in delivered dose. In prostate cancer, there is evidence that small, yet vital, structures, such as the prostatic urethra,^58^ neurovascular bundles, and internal pudendal arteries, play a role in modulating toxicity. There are uncertainties in urethral contouring, which can be largely mitigated through a standardized contouring approach using MRI^59^ and reproducible daily positioning. Although urethral dose has not been the primary endpoint of a randomized clinical trial with efficacy or toxicity outcomes to date, Leeman et al^16^ demonstrated in a study-level meta-analysis the correlation of urethral dose to moderate and high-grade urinary toxicity with SBRT. On balance, the use of a urethral PRV is gaining wider acceptance in this setting, as demonstrated by its inclusion as an OAR in current prostate SBRT clinical trials such as TROG 18.01 Novel Integration of New prostate radiation therapy schedules with adJuvant Androgen deprivation,^60^ Thus, methods to more closely approximate the delivered dose on the dynamic anatomy to the planned dose, such as KIM, may help to minimize urinary toxicity.

More recently, there has been a concerted effort to identify drivers of erectile dysfunction with prostate RT. Although RT may impact nerve function, the primary driver of erectile dysfunction may be more related to the impact on vascular function. This was first tested by Spratt et al^14^ in a single-arm phase 2 trial of patients receiving “vessel sparing” dose-escalated external beam RT or combination brachytherapy with variable use of androgen deprivation therapy. Vessel sparing entails reducing dose to protect the neurovascular bundles, internal pudendal arteries, and penile bulb. At 5 years after treatment, nearly 90% of patients remained sexually active. This finding was unprecedented and motivated the conduct of a randomized trial, prostate oncologic therapy ensuring neurovascular conservation, which recently reported its interim results.^15^ Although the primary endpoint is 2-year posttreatment quality of life, at 3 months, there were trends in improvement in sexual function (*P* = .096) and significant improvements in urinary irritative/obstructive domain (*P* = .02).

### Improved dominant nodule boosting

Precisely, sparing small OARs and accurately tracking intraprostatic motion becomes even more critical when employing focal or microboosting strategies for dominant intraprostatic lesions (DILs). The typical boosting dose applied to DILs, often 45 Gy delivered in 5 fractions, frequently exceeds dose constraints for nearby OARs, underscoring the necessity of stringent dose tracking and delivery precision. The ability of real-time IGRT to maintain the planned dose in the presence of motion, and the impact of DILs being underdosed if motion is not managed, has been shown.^17^

### Enable fewer fraction treatments to improve health economics, the patient experience, and sustainability

Over the last 15 years, successive phase 3 trials have sequentially shown that RT schedules can be shortened from 8 to 4 weeks and, latterly, to just 5 to 7 fractions in total.^61,62^ The next logical question is whether patients can be cured, without increased side effects, in fewer than 5 fractions. Several trials are currently investigating the safety and feasibility of 2-fraction SBRT, including randomized trial of five or two MRI-guided adaptive radiotherapy treatments for prostate cancer (NCT04984343), improving sexual quality of life - randomized trial of two vs five MRI guided SABR treatments for prostate cancer (NCT05600400), and two-fraction versus five-fraction stereotactic radiotherapy for localized prostate cancer (NCT06027892).

The single-center, phase 2 randomized hypofractionated expedited radiotherapy for men with localisEd proState cancer (NCT04595019) trial to our knowledge, was the first to report acute toxicity outcomes (the primary endpoint). Fortysix patients with localized prostate cancer were randomized to MRI-guided adaptive SBRT, with either 5 fractions (36.25 Gy in 5 fractions; 40 Gy to CTV) or 2 fractions (24 Gy in 2 fractions; 27 Gy in 2 fractions to macroscopic boost). Although not powered to be comparative, the rates of Common Terminology Criteria for Adverse Events grade 2 or higher genitourinary toxicity appeared similar in those treated in 5 versus 2 fractions (29.2% vs 27.3%). No grade ≥2 acute gastrointestinal toxicity was seen in the 2-fraction group.^18^ These encouraging data need to be validated in larger studies and should be able to show noninferiority of biochemical and toxicity outcomes.

The number of patients diagnosed with prostate cancer is set to double between 2020 and 2040 when 2.9 million people per year are estimated to be affected. In a world where most countries have insufficient linacs even for existing demand, there is a strong imperative to maximize the number of patients who can be treated by each linac and staff member. SBRT is expected to be a critical component of making best use of these scarce resources. Shorter schedules also play a role in reducing the carbon footprint of RT.^19^

### Increased correlation between dose delivered and treatment outcomes, and improved data for dose-response modeling

Unaccounted for anatomic changes that occur during and between fractions change the dose distribution delivered to the patient from that planned. Aligned with the Quantitative Analysis of Normal Tissue Effects in the Clinic article describing the importance of accurate dose accumulation in patients,^20^ there is evidence that delivered dose is a better indicator of clinical response for pelvic RT than planned dose.^21,22^ The SPARK trial^13^ showed, by using a delivered dose accumulation tool^41^ that accounts for intrafraction motion that the estimated dose being delivered to the patient with real-time IGRT is significantly closer to the planned dose with motion monitoring and gated or MLC adaptation than the standard of care without real-time IGRT. This finding means that, with real-time IGRT, not only are the patient dose distributions improved, with higher target dose and more consistent normal tissue dose, but there is also less uncertainty in delivered dose, meaning that biological responses, such as those for prostate SBRT in the Hypofractionated Treatment Effects in the Clinic special issue,^23,24^ will have less uncertainty. As the target coverage with real-time IGRT is higher than without real-time IGRT from which much of the dose-response understanding has been acquired, the required prescription dose with real-time IGRT may be lower than without real-time IGRT.

### Lessons learned

Reflecting on the translational journey of KIM leads to 3 main lessons learned of the successful elements that could assist others developing similar bench-to-bedside translational research: determination and belief, collaboration with attitude, and serendipity. Determination and belief are needed to dedicate significant parts of many people’s professional careers from 2007, when KIM was a mathematical concept, to experiments, to clinical trials, and on its present track to a product (Fig. 3). Underpinning the determination and belief is the clinical driver that our patients expect—that we know where the cancer target is when we are delivering high doses of radiation to achieve the benefits shown in Figure 1.

Collaboration with attitude is the realization that many diverse people across geographic, professional, and commercial boundaries have significantly contributed to the KIM program. The attitude from the outset has been a focus on “how do we make this device work effectively and safely in the clinic.” A committed team with an outcome-focused attitude was essential to success at each of the project stages shown in Figure 3.

Serendipity comes from many factors, finding clinicians willing to trust part of the care of their patients to untested but promising technology, succeeding in obtaining competitive grant funding, running clinical trials in a jurisdiction (Australia) where the barriers to medical device trials are lower than in other countries, and developing technology at a time where there was increasing interest in and recommendations for deployment including AAPM^1^ and American Society for Radiation Oncology^2^ guidelines. The team had varying degrees of control over these outcomes. However, luck and timing played an important role.

### Looking forward

Looking forward, KIM is being explored in other body locations such as liver cancer,^63,64^ availability through commercialization with an industry partner, and through a 300-patient 10-center clinical trial. This Prostate Radiation Therapy Kilovoltage Intrafraction Monitoring Real-World Expansion multicenter feasibility trial (NCT03561961) will evaluate KIM’s impact on workflow and workload by implementing KIM monitoring across networked GenesisCare sites. The primary endpoint is the proportion of successful treatment sessions defined as completion within 20 minutes with uninterrupted real-time tracking. Secondary objectives include retrospective assessment of precision and accuracy in broad deployment, quality-of-life outcomes, gating frequency, correlation with patient and machine factors, and data collection to support the development of marker-less tracking.

## Conclusion

SBRT schedules for prostate cancer provide high efficacy in clinical trials but require additional efforts at accurate treatment delivery to maximize the therapeutic ratio in the community setting. KIM is a technology developed to address the unmet clinical need for accurate intrafraction motion management without the need for additional hardware on standard linear accelerators. The collaboration between researchers, clinicians, and industry has led to KIM being developed from a concept to clinical trials, enabling the safe delivery of prostate cancer SBRT in the presence of intrafraction motion. This journey provides a template for similar bench-to-bedside translational research.

## Data Availability

To enable the clinical and scientific community to maximize the utility of the data that the patients and trial team took so much time and effort to acquire and curate, the TROG 15.01 SPARK (Stereotactic Prostate Adaptive Radiation therapy using Kilovoltage Intrafraction Monitoring) clinical trial data set has been made open source (https://doi.org/10.25910/qg5d-6058).
